# Senolytic Peptide FOXO4-DRI Selectively Removes Senescent Cells From *in vitro* Expanded Human Chondrocytes

**DOI:** 10.3389/fbioe.2021.677576

**Published:** 2021-04-29

**Authors:** Yuzhao Huang, Yuchen He, Meagan J. Makarcyzk, Hang Lin

**Affiliations:** ^1^Department of Orthopaedic Surgery, University of Pittsburgh School of Medicine, Pittsburgh, PA, United States; ^2^Department of Orthopaedics, The Third Xiangya Hospital, Central South University, Changsha, China; ^3^Department of Bioengineering, University of Pittsburgh Swanson School of Engineering, Pittsburgh, PA, United States; ^4^McGowan Institute for Regenerative Medicine, University of Pittsburgh School of Medicine, Pittsburgh, PA, United States

**Keywords:** chondrocyte, senescence, autologous chondrocyte implantation, senolytic, FOXO4-DRI

## Abstract

Autologous chondrocyte implantation (ACI) is a procedure used to treat articular cartilage injuries and prevent the onset of post-traumatic osteoarthritis. *In vitro* expansion of chondrocytes, a necessary step in ACI, results in the generation of senescent cells that adversely affect the quality and quantity of newly formed cartilage. Recently, a senolytic peptide, fork head box O transcription factor 4-D-Retro-Inverso (FOXO4-DRI), was reported to selectively kill the senescent fibroblasts. In this study, we hypothesized that FOXO4-DRI treatment could remove the senescent cells in the expanded chondrocytes, thus enhancing their potential in generating high-quality cartilage. To simulate the *in vitro* expansion for ACI, chondrocytes isolated from healthy donors were expanded to population doubling level (PDL) 9, representing chondrocytes ready for implantation. Cells at PDL3 were also used to serve as the minimally expanded control. Results showed that the treatment of FOXO4-DRI removed more than half of the cells in PDL9 but did not significantly affect the cell number of PDL3 chondrocytes. Compared to the untreated control, the senescence level in FOXO4-DRI treated PDL9 chondrocytes was significantly reduced. Based on the result from standard pellet culture, FOXO4-DRI pre-treatment did not enhance the chondrogenic potential of PDL9 chondrocytes. However, the cartilage tissue generated from FOXO4-DRI pretreated PDL9 cells displayed lower expression of senescence-relevant secretory factors than that from the untreated control group. Taken together, FOXO4-DRI is able to remove the senescent cells in PDL9 chondrocytes, but its utility in promoting cartilage formation from the *in vitro* expanded chondrocytes needs further investigation.

## Introduction

Autologous chondrocyte implantation (ACI) is a biomedical treatment that repairs cartilage injury in the knee joint, which has been shown to reduce pain and facilitate mobility recovery (Kreuz et al., 2019). It has also been reported that an ACI procedure may prevent the progression of early osteoarthritis in patients with cartilage injuries (Jungmann et al., 2019). However, it is noted that the clinical outcome of ACI is still variable (Andriolo et al., 2017), and the reasons have not been fully demonstrated. Given that implanted chondrocytes are responsible for the generation of new cartilage in the defect site, it is not surprising that the quality of these cells critically affects the reparative results of ACI treatment (Davies and Kuiper, 2019).

Typically, ACI involves harvesting cartilage, isolating chondrocytes, expanding cells *in vitro*, and re-implanting over an articular cartilage defect. In order to collect a sufficient number of cells for implantation, isolated chondrocytes usually undergo an extensive expansion from 0.1–0.2 million to 40–60 million (Huang et al., 2016). During this period, cultured chondrocytes gradually lose the proliferative capacity and the potential of generating the cartilage-specific matrix, a phenomenon known as dedifferentiation (Sassi et al., 2014). Moreover, the number of senescent cells also increase with culture time, resulting in a loss of division capabilities, resistance to apoptosis, and the acquisition of a robust proinflammatory secretome known as the senescence-associated secretory phenotype (SASP) (Jeon et al., 2018). The representative SASP factors include pro-inflammatory cytokines, chemokines, and proteases, which can cause an imbalance in cartilage hemostasis, resulting in degradation or other dysfunctions (Li and Pei, 2012). In fact, transplanting senescent cells into mice knee joint has been found to induce an osteoarthritis-like change (Xu et al., 2017).

Thus, selectively removing senescent cells in chondrocytes is crucial to assure the quality of cells for ACI. Currently, several senolytics were reported to kill senescent cells (Di Micco et al., 2021). FOXO4-DRI is a peptide antagonist designed to perturbs the interaction of FOXO4 and p53. Disrupting the p53-FOXO4 interaction causes p53 to be excluded from the nucleus and directed to mitochondria for induction of apoptosis in senescent cells, ultimately eliminating senescent fibroblasts through triggering apoptosis (Baar et al., 2017). Whether this novel senolytic can remove senescent chondrocytes, thus finally enhancing the reparative outcome of ACI has not been reported.

In this study, we tested the hypothesis that FOXO4-DRI treatment can selectively remove senescent cells in expanded human chondrocytes, thus enhancing their potential to generate high-quality cartilage. Specifically, to simulate the *in vitro* expansion for ACI, human chondrocytes were expanded to a population doubling level (PDL) 9 to represent chondrocytes ready for implantation. Cells at PDL3 were used as the minimally expanded control. The senescent levels of PDL3 and PDL9 chondrocytes were assessed by senescence-associated beta-galactosidase (SA-β-gal) staining and qRT-PCR analysis of senescence-associated marker genes. Then both PDL3 and PDL9 chondrocytes were treated with FOXO4-DRI for 5 days. Afterward, cell phenotype was examined by testing their senescence level and cartilage formation capacity.

## Materials and Methods

### Isolation and *in vitro* Expansion of Human Chondrocytes

Human chondrocytes were isolated from de-identified knee articular cartilage without signs of osteoarthritis or injury. Tissues were obtained through the National Disease Research Interchange (Philadelphia, PA) with approval from the University of Pittsburgh Committee for Oversight of Research and Clinical Training Involving Decedents (CORID). To isolate chondrocytes, fresh articular cartilage tissues were rinsed with basal medium (BM), consisted of high glucose Dulbecco’s modified Eagle’s medium (DMEM, Gibco/Thermo Fisher Scientific, Waltham, MA, United States), with 2 × Antibiotics-Antimycotics (anti-anti, Life Technologies, Carlsbad, CA, United States) and then cut into ∼1 mm^3^ pieces. The cartilage particles were digested with collagenase type II (0.2% w/v in BM, Worthington Biochemical, Lakewood, NJ, United States) in a 37°C shaker overnight. After passing through the 70 μm cell strainer (BD Falcon, Bedford, MA, United States), the cell suspension was centrifuged at 300 × g for 5 min. Cells resuspended in the growth medium (GM, DMEM containing 10% fetal bovine serum (FBS, Life Technologies) and 1% anti-anti), and the single cells were seeded in a cell culture flask. GM was replaced every 3 days. After chondrocytes reached 80–90% confluency, the cells were dissociated with Trypsin/EDTA (Gibco/Thermo Fisher Scientific) and passaged. The chondrocytes used in this study were isolated from eight donors (15-, 70-, 73-years-old female, and 42-, 69- and 70-years-old male) without the sign of cartilage degradation. For our studies, chondrocytes were expanded *in vitro* in GM and incubated at 37°C with 5% CO_2_. Chondrocytes were passaged at 80–90% confluency.

### Population Doubling Level Calculation

To calculate the PDL, a formula was used as follows: **n** = 3.32 (log **UCY** −log **I**) + **X**, where **n** is the final PDL at the end of a given subculture, **UCY** is the cell yield at that point, **I** is the cell number used as inoculum to begin that subculture, and **X** is the PDL of the inoculum used to initiate the subculture being quantitated (Pham et al., 2020). According to the formula, both UCY and I are recorded after each passage. Cell number was assessed by hemocytometer counting.

### SA-β-Galactosidase Staining

Cells were seeded in 6-well microplates at 4 × 10^4^ cells/well. Cellular senescence was assessed by SA-β-galactosidase staining (SA-β-gal staining) using senescence β-galactosidase Staining Kit (Cell Signaling Technology, Beverly, MA, United States) according to the manufacturer’s instructions. Cells stained blue under an optical microscope were counted as positive cells, and DAPI counterstain was used to count the total cell number. The percentage of SA-β-gal staining-positive cells was calculated in at least 3 random fields.

### CCK-8 Assay

Chondrocytes were plated at 3 × 10^3^ cells/cm^2^ in 24-well microplates with growth medium. The ability of proliferation was assessed by a Cell Counting Kit-8 (CCK-8, Dojindo, Rockville, MD, United States) at different time points post-plating. For counting, each condition was plated in triplicate. At each time point, 1/10 volume of culture medium per well CCK-8 solution reagent was added, incubated at 37°C for 3 h, and the absorbance was measured at 450 nm using a microplate reader.

### RNA Extraction and qRT-PCR Detection

Chondrocytes were plated at 3 × 10^3^ cells/cm^2^ in 6-well microplates (monolayer culture) with GM after treatment. Total RNA was harvested from monolayer and pellet cultures at different time points using Qiazol (Qiagen, Germantown, MD, United States) and purified with RNeasy Plus Universal Mini Kit (Qiagen, Cat. No. 74104) according to the manufacturer’s protocol. Then cDNA was synthesized using SuperScript^TM^ IV VILO^TM^ Master Mix (Invitrogen, Waltham, MA, United States) and diluted 10–1 by DNase/RNase-Free Distilled Water (Invitrogen). A 20 μl mix, containing 8 μl of cDNA sample, 10 μl of SYBR Green PCR Master Mix (Thermo Fisher Scientific), and 2 μl of 10 mM forward primer and reverse primer mixture was used to performed Quantitative RT-PCR with a StepOne Plus Real-time PCR system (Applied Biosystems, Foster City, CA, United States). The relative gene expression was calculated using the 2^–Δ*CT*^ method by Ribosomal protein L13a (*RPL13*α) gene, and the fold change was expressed by 2^–ΔΔ*CT*^. For all real-time RT-PCR experiments, each condition was in triplicate. Full name and abbreviation of genes and proteins tested in this study are shown in Supplementary Table 1. Sequences of primers for qRT-PCR are listed in Supplementary Table 2.

### Drug Treatment

In this study, we used FOXO4 D-Retro-Inverso peptide (FOXO4-DRI, NovoPro, Shanghai, China) and ABT-263 (Apexbio Technology, Houston, TX, United States) to selectively remove the senescent chondrocytes. The final concentration of FOXO4-DRI and ABT-263 was 25 and 1.25 μM in 2% FBS basal medium. The duration of treatment was 5 days. After treatment, cells were maintained in GM for 2–3 days before analysis.

### Caspase-3/7 Staining

5 × 10^3^ cells/cm^2^ chondrocytes were plated in 24-well microplate and cultured with GM. Fluorescent staining for casepase3/7 was done using CellEvent^TM^ Caspase-3/7 Green Detection Reagent Kit (Thermo Fisher Scientific). Diluted reagent was added to cells after treatment and incubated for 30 min at 37°C. Cells were viewed under a fluorescence microscope. Apoptotic cells appeared as bright green, while non-apoptotic cells did not show any signal.

### Western Blot

Total proteins were extracted in RIPA buffer (Sigma-Aldrich) that was supplemented with protease inhibitor cocktail (100× Sigma-Aldrich). The protein concentration was measured by BCA kit (Thermo Scientific^TM^ Pierce^TM^ BCA Protein Assay Kit, Waltham, MA, United States). The samples were diluted in RIPA, mixed with Laemmli buffer (Bio-Rad, Hercules, CA), and then denatured at 95°C for 5 min. Proteins were fractionated electrophoretically on NuPAGETM 4–12% Bis-Tris Polyacrylamide Gel (Invitrogen) and then transferred to a polyvinylidene fluoride (PVDF) membrane using the iBlot Dry Blotting System (Invitrogen). The membrane was blocked with 3% non-fat milk (BioRad, Hercules, CA, United States) at room temperature for 1.5 h, washed, and incubated with primary antibodies (Supplementary Table 3) at 4°C overnight on a rotating shaker. On the next day, after the membrane was washed with TBST for five times, HRP-conjugated secondary antibody (Thermo Scientific) was applied for 1.5 h at room temperature. Finally, the membrane was incubated in SuperSignal West Dura Extended Duration Substrate (Thermo Fisher Scientific Pierce Protein Biology) and imaged with ChemiDocTMTouch Imaging system (BioRad).

### Three-Dimensional Pellet Culture of Chondrocytes

For the 3D pellet culture of chondrocytes, 2.5 × 10^5^ cells were used to form one pellet. Cells were seeded in 96-well MicroWell round-bottom plates (Thermo Fisher Scientific). And then the plate was centrifuged at 300 g for 10 min to condense chondrocytes. The medium used to maintain the pellet was full chondrogenic medium (CM) consisted of DMEM (Gibco), 2× anti-anti (Gibco), 10 μg/ml ITS (Thermo Fisher Scientific), 0.1 μM Dexamethasone (Sigma-Aldrich, St. Louis, MO, United States), 40 μg/ml Proline (Sigma-Aldrich), 50 μg/ml ascorbic acid (Sigma-Aldrich) and 10 ng/ml TGF-β1 (Peprotech, Rocky Hill, NJ, United States). Each pellet was cultured in 200 μl full CM and incubated at 37°C with 5% CO_2_. CM was replaced every 48 h. After 14 days of culture, pellets were harvested for histological analysis.

### Safranin O Staining

Pellets were fixed overnight at 4°C in 10% buffered formalin (Fisher Chemical, Hampton, NH, United States), dehydrated in gradient ethanol, cleared in xylene, and then embedded in paraffin. The blocks were sectioned at 6 μm thickness using a Leica microtome (Model RM 2255). For Safranin O/Fast Green staining, slides were stained with hematoxylin (Mayer, Sigma) for 5 min, 0.005% Fast Green (Thermo Fisher Scientific, Pittsburgh, PA, United States) for 1 min, rinsed with 1% acetic acid, and stained with 0.1% Safranin O (Millipore, Billerica, MA, United States) for 20 min. Histological staining was imaged using a microscope equipped with a color digital camera (Nikon Eclipse E800).

### Statistical Analysis

Each experiment was repeated three or more times with three experimental replicates, and the results were expressed as the mean ± SD. Graphpad Prism 8 (GraphPad Software, San Diego, CA, United States) was applied for statistical analysis. Significant differences among different groups were determined by two-tailed Student’s *t*-test for two-group comparisons. One-way or two-way analysis of variance (ANOVA) was used for multiple-group comparisons. Statistical significance was considered at *p* < 0.05.

## Results

### Extensive *in vitro* Chondrocyte Expansion Results in the Generation of Senescence Cells

**FIGURE 1:**
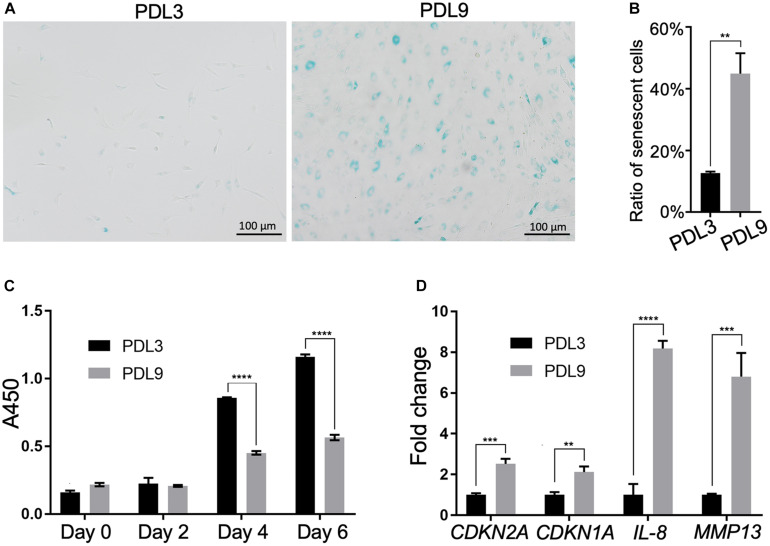
Comparison of senescent level between PDL3 and PDL9 chondrocytes. **(A)** SA-β-gal staining. Bar = 200 μm. (**B**) Ratios of senescent cells. **(C)** CCK-8 assay was used to assess cell proliferation potential. **(D)** Expression levels of senescence-relevant genes. The data was normalized to that in PDL3 group (set as 1). **p < 0.01; ***p < 0.001; ****p < 0.0001.

We first characterized cells in the early passage (PDL3, 0.1 million cells were expanded to ∼0.8 million cells) and late passage (PDL9, 0.1 million cells were expanded to ∼50 million cells), which, respectively, represented the minimally expanded chondrocytes and chondrocytes ready for ACI. As shown in Figures 1A,B, there were more SA-β-gal staining-positive chondrocytes in PDL9 group (>40%) than PDL3 group (<17%). It should be noted that the staining intensity was also higher in the PDL9 group.

Level of senescence was further examined by testing the proliferative ability and expression of SASP genes. Initially, PDL3 and PDL9 cells were seeded at the same density. After 2, 4, and 6 days of culture, the cell number in the PDL9 group was significantly lower than that in the PDL3 group (Figure 1C), which indicated that the proliferative ability of senescent chondrocytes was significantly decreased. qRT-PCR analysis revealed the marked upregulation of *CDKN2A (encoding p16)*, *CDKN1A (encoding p21)*, interleukin-8 (*IL-8)*, and matrix metalloproteinase (*MMP)-12* expression (Figure 1D). Taken together, PDL9 chondrocytes contained significantly more senescent cells than PDL3 chondrocytes, confirming that extensive *in vitro* expansion led to chondrocyte senescence.

### FOXO4-DRI Treatment Removes Cells in PDL9 Chondrocyte Culture

**FIGURE 2:**
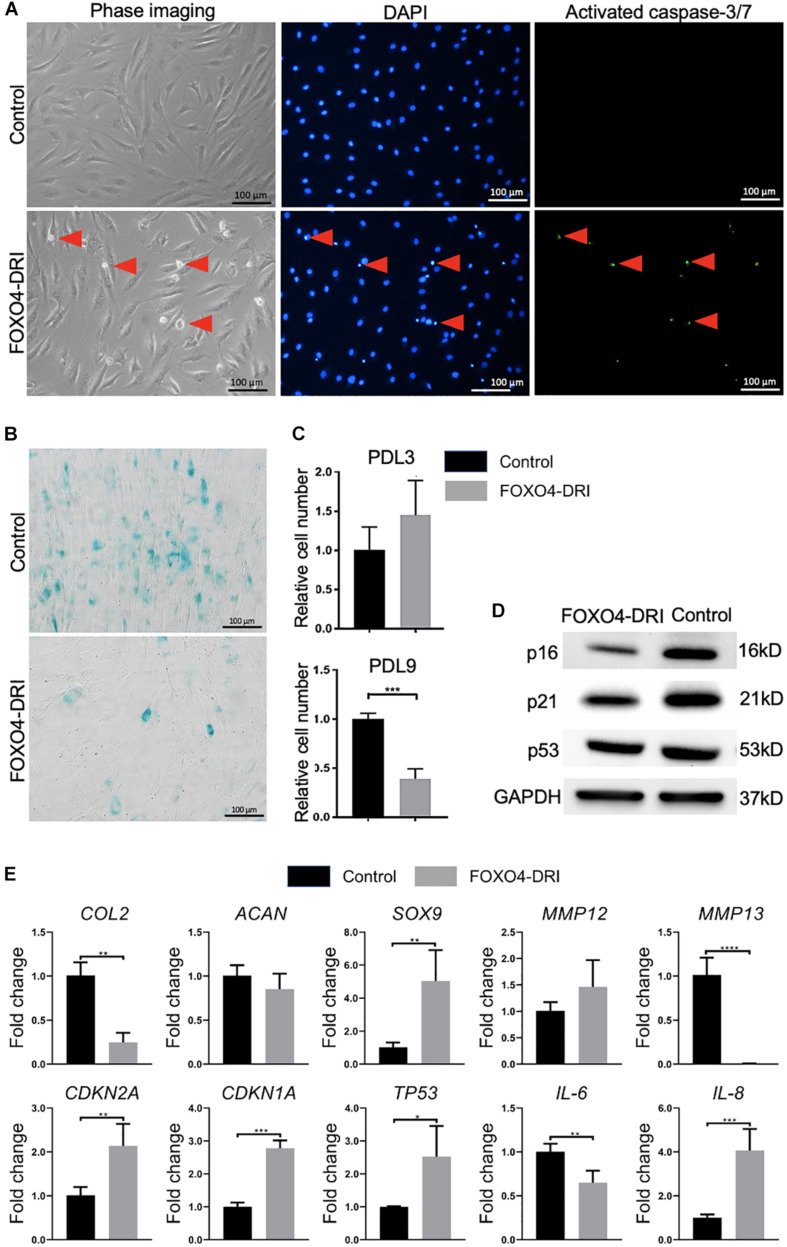
Assessment of senescence level in PDL9 cells after FOXO4-DRI treatment. The samples with (FOXO4-DRI) or without (Control) FOXO4-DRI treatment were analyzed. **(A)** Detection of activated caspase-3/7. Red arrows indicate apoptotic cells. Bar = 100 μm. **(B)** SA-β-gal staining. Bar = 100 μm. **(C)** The relative cell number in PDL3 and PDL9 chondrocytes after FOXO4-DRI treatment was assessed using MTS assay. **(D)** Western blot. **(E)** Expression levels of selective genes. The data was normalized to that in Control group (set as 1). *p < 0.05; **p < 0.01; ***p < 0.001; ****p < 0.0001.

We next test the efficacy of the previously reported senolytics, including ABT263 and FOXO4-DRI, in removing senescent chondrocytes. As shown in Supplementary Figure 1, we did not observe a significant difference in the cell number between the control and ABT263 treatment group. In contrast, FOXO4-DRI treatment significantly reduced the cell number in the PDL9 group (Supplementary Figures 1, 2). Interestingly, FOXO4-DRI did not affect cell viability in PDL3 group (Supplementary Figures 1, 2). Results from Figure 2A confirmed that FOXO4-DRI killed the cells through inducing apoptosis.

### FOXO4-DRI Treatment Significantly Reduces the Senescence Level in PDL9 Cells

SA-β-gal staining (Figure 2B) indicated that FOXO4-DRI treatment led to a significant decrease in the number of senescent cells in the PDL9 group (<5%). qRT-PCR analysis (Figure 2E) showed that FOXO4-DRI-treated PDL9 cells displayed enhanced *SOX9* expression, and reduced *MMP12* and *MMP13* expression, which however were at the expense of lower expression levels of *COL2*. We also observed a reduction in *IL-6* expression and an increase in *IL-8* expression after FOXO4-DRI treatment. Furthermore, western blot was used to analyze the protein levels of representative senescent markers, including p16, p21, and p53. A significant decrease of these marker proteins was observed in FOXO4-DRI treated group compared with the control group (Figure 2D). However, the gene expression levels of these molecules displayed an opposite trend (Figure 2E). Taken together, FOXO4-DRI is capable of selectively removing senescence cells in PDL9 chondrocytes.

### FOXO4-DRI Treatment Does Not Markedly Improve the Regenerative Potential of PDL9 Chondrocytes

**FIGURE 3:**
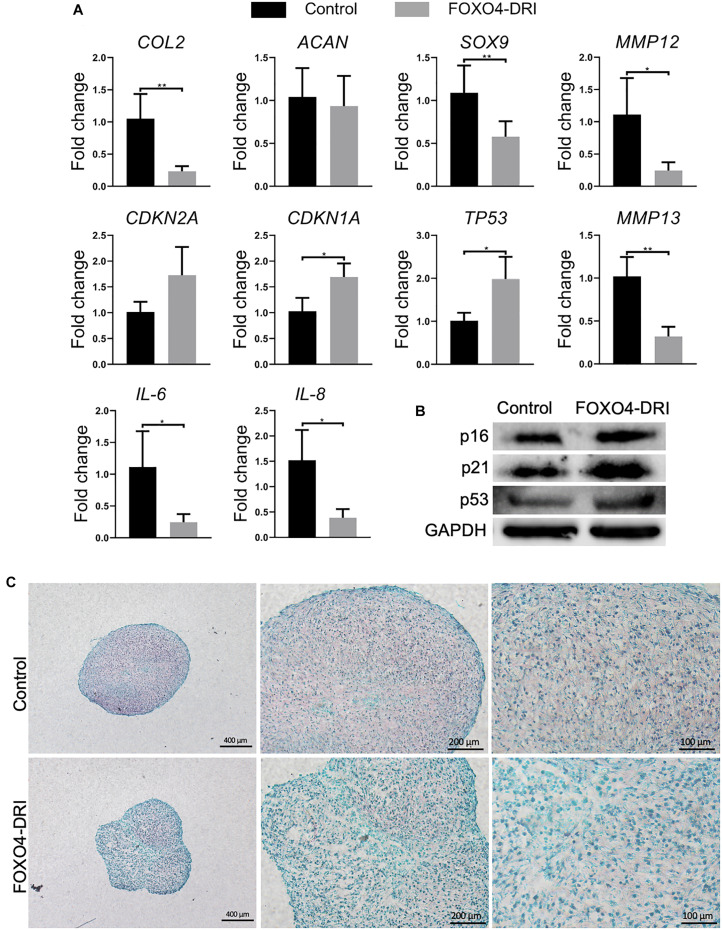
Assessment of senescence level and chondrogenesis in engineered cartilage. PDL9 were pretreated with (FOXO4-DRI) or without (Control) FOXO4-DRI for 5 days and then subjected to 14 days of chondrogenic cultures. **(A)** Expression levels of selective genes. The data was normalized to that in the Control group (set as 1). **(B)** Western blot. **(C)** Safranin O/Fast green staining. Bar = 400, 200, and 100 μm from the left to right. *p < 0.05; **p < 0.01.

The regenerative potential of PDL9 chondrocytes after FOXO4-DRI treatment was assessed by 3D pellet culture. As shown in Figure 3A, reduced expression levels of *IL-6 and 8* and *MMP3, 12 and 13* were observed in the FOXO4-DRI treated group, which however were concomitantly accompanied with reduced expression of *SOX9* and *COL2*, as well as increased expression of *CDKN2A, CDKN1A*, and *TP53 (encoding p53)*. Western blot analysis further showed that cartilage generated from FOXO4-DRI pre-treated chondrocytes displayed higher protein levels of p16, p21, and p53 (Figure 3B). In addition, we did not observe increased deposition of GAG from FOXO4-DRI pre-treated PDL9 chondrocytes (Figure 3C).

## Discussion

ACI has been shown to regenerate new tissues, reduce pain, and increase joint function after cartilage injury. However, the clinical outcomes from ACI are not always efficacious or consistent. Given that implanted chondrocytes are major cells that account for creating new tissues, their quality may directly affect the phenotype and function of regenerated cartilage. Surprisingly, no established criteria were available to comprehensively characterize the expanded chondrocytes before they are implanted. In this study, we aimed to analyze senescence during chondrocyte expansion and use senolytics to remove senescent chondrocytes for the enhancement of cartilage formation in ACI.

The loss of the chondrocytic phenotype and proliferative potential during *in vitro* expansion, termed as “dedifferentiation,” has been well documented. Chondrocyte dedifferentiation was majorly characterized as the reduced expression of COL2 and ACAN and enhanced expression of type I collagen and versican (Charlier et al., 2019). However, the association between reduced chondrocyte quality during expansion and ACI outcome is less studied. Pietschmann et al. (2009) sampled the newly formed cartilage after ACI and suggested that the number of morphologically abnormal cells was correlated with a poor clinical outcome. Niemeyer et al. (2012) moved one step forward by saving some cells from ACI and studying the correlation between chondrocyte phenotype before implantation, and clinical outcome after 6, 12, and 24 months. Results from this study indicated that the postoperative International Knee Documentation Committee (IKDC) score was significantly influenced by *COL2* expression, but not *ACAN* (Niemeyer et al., 2012). Recently, a cell identity assay was employed to assess the contamination of human chondrocytes by human synovial fibroblasts. The results showed a higher cell identity score, meaning less fibroblast contamination, and was significantly correlated with structural repair quality and graft survival after ACI (Ackermann et al., 2019). These results collectively signified the importance of enhancing chondrocyte quality for the improvement of final clinical outcomes.

In addition to dedifferentiation, another consequence of *in vitro* expansion is replicative senescence, which also significantly influences chondrocyte function. The observation of senescent cells in *in vitro* expanded chondrocytes have been reported as early as 2002. In this study, articular chondrocytes were expanded to a number that was needed for ACI. These chondrocytes displayed a telomere erosion in the range of 900 bp, which was comparable with 30 years of native aging (Parsch et al., 2002). In our study, significantly more SA-β-gal staining-positive cells were observed in PDL9 chondrocytes. Similar results were reported in a recent study (Ashraf et al., 2016). Interestingly, passage 2 and 6 chondrocytes were used, which were roughly equivalent to the PDL3 and PDL9 cells used in our current study. Our results also indicate that senescent chondrocytes present a larger and flatter cell shape, which is consistent with a previous study (Cha et al., 2013).

The detrimental influence of senescence has been previously demonstrated. They negatively influence neighbor cells by secreting SASP (Borodkina et al., 2018). In fact, it has been shown that the translation of senescence chondrocytes in mice knee joint resulted in OA phenotype, characterized by leg pain, impaired mobility, and radiographic and histological changes (Xu et al., 2017). These studies signify the importance of removing the senescence cells in enhancing reparative outcomes from ACI.

In our study, ABT 263 (or UBX0101) was first tested because of its reported capacity in clearing senescent cells. For example, chondrocytes isolated from OA cartilage displayed significant senescence levels, and ABT263 treatment reduced the expression of inflammatory cytokines and promoted cartilage matrix aggregation in OA chondrocytes (Yang et al., 2020). Similar results were found in the radiation-induced senescent chondrocytes (Jeon et al., 2017). It should be noted that expression of p16 actually increased after 3 days of ABT 263 treatment, indicating the potential stress of senolytics on cells. Moreover, although the expression of p21 decreased after treatment, the protein level was higher in cells treated with ABT 263 than the untreated control. Regarding the chondrogenic potential, results from both studies confirmed that the removal of senescent cells significantly increased the cartilage formation with less production of degrative enzymes. However, similar senolytic potential of ABT 263 was not seen in our study. Of note, the senescence chondrocytes tested here were induced by replication stress. In previous studies, senescence chondrocytes were either from OA cartilage, or induced by other methods.

Next, we tested the potential of FOXO4-DRI in removing senescent chondrocytes, which has not been reported before. Mechanistically, the peptide perturbs the FOXO4 interaction with p53, which selectively causes p53 nuclear exclusion and cell-intrinsic apoptosis (Baar et al., 2017). As demonstrated in Figure 2, this novel senolytic did remove cells from the PDL9 chondrocytes, which was accompanied by significantly reduced levels of SA-β-gal staining. Surprisingly, there was not a noticeable cell loss in the culture of non-senescent PDL3 chondrocytes, suggesting the selective potential of FOXO4-DRI. Since it is suggested that p53, p16, p21 are directly related to the induction of senescence in chondrocytes (Ashraf et al., 2016), we thus used qRT-PCR and western blot analysis to examine their expression levels. We observe the reduction of both protein and RNA levels of all these three molecules. We also analyzed the expression of *IL-6* and *-8*, which however were not detectable due to the low expression level. Again, in the first study using FOXO4-DRI to induce apoptosis in senescent cells, the senescent cells were generated by irradiation or doxorubicin (Baar et al., 2017). In a recent study, FOXO4-DRI decreased levels of p53, p21, and p16 in the testes of aged mice, when compared to young mice (Zhang et al., 2020). Therefore, our study and published work collectively showed that FOXO4-DRI was able to remove senescent cells created by different methods.

As shown in Figure 3, FOXO4-DRI pre-treatment is the dedifferentiation of chondrocytes during the *in vitro* expansion. Although not all cells in PDL9 were senescent cells, the intrinsic chondrogenic potential has been impaired during the dedifferentiation process. In previous studies that compared the differentiation capacity of chondrocytes from different passages, the results indicated that cells at early passages usually generated more cartilage matrix than those at late passages upon chondroinduction (Schulze-Tanzil et al., 2002; Ma et al., 2013). Therefore, the treatment with senolytics may not be able to reverse the loss of chondrogenic potential in PDL9. However, the new cartilage created by FOXO4-DRI pretreated PDL9 cells indeed displayed reduced expression of several SASP factors, implying the benefit of FOXO4-DRI pre-treatment in enhancing the cartilage quality. Surprisingly, increased p21 level was observed in the FOXO4-DRI pre-treatment groups, suggesting senolytics might be a potential stressor to cells. Similar results were reported in a previous study, which used another type of senolytic, ABT263, to treat chondrocytes isolated from osteoarthritic cartilage (Yang et al., 2020). Therefore, the potential adverse influence of senolytics needs to be further investigated.

There are several limitations in the current study. First, we only examined limited senolytics. Also, we used the doses that were reported in a previous study, which may not be optimal for treating PDL9 chondrocytes. In the future, we will test more senolytics with different doses. Recently, oleuropein, a polyphenol, was shown to have the senolytic potential in OA chondrocytes (Varela-Eirín et al., 2020). Whether it is able to selectively remove senescent chondrocytes created by the *in vitro* expansion requires additional study. Second, the mechanistic analysis was not performed to explain why removing senescent cells did not result in more cartilage formation. Third, the combination of senolytics with other methods for reducing senescence was not examined. Recently, extracellular matrix derived from mesenchymal stem cells were shown to maintain re-differentiation potential of human chondrocytes (Pei and He, 2012; Yang et al., 2018). Also, the low-oxygen environment has been found to suppress senescence (Moussavi-Harami et al., 2004). These methods can be adapted to the current study to increase the potential of senolytics in enhancing ACI. Lastly, the strategy to augment the chondrogenic potential of senolytics-treated chondrocytes needs to be explored. For example, conventional chondrogenic factors (Jonitz et al., 2012), epigenetic manipulation with specific agents (Duan et al., 2017), or co-culture with mesenchymal stem cells (Lai et al., 2013), have been shown to promote or maintain chondrocytic phenotype. These methods can be used either during or after the senolytic treatment.

## Conclusion

*In vitro* expanding human chondrocytes to a number that is required by the ACI results in the generation of senescent cells. Although FOXO4-DRI treatment did remove the senescent cells, the cartilage formation capacity of retained chondrocytes was not improved. Of note, cartilage created by FOXO4-DRI pretreated chondrocytes displays a lower senescence level than that from the untreated counterparts. Our results indicate the utility of FOXO4-DRI for removing senescent chondrocytes. In the future, other methods are needed to restore the chondrogenic potential of retained chondrocytes after FOXO4-DRI treatment.

## Data Availability Statement

The original contributions presented in the study are included in the article/Supplementary Material, further inquiries can be directed to the corresponding author/s.

## Author Contributions

HL conceived the study. YZH and YH designed and carried out all the experiments. YZH, YH, and HL analyzed the results. YZH, MM, and HL wrote the first draft. All authors reviewed and edited the manuscript.

## Conflict of Interest

The authors declare that the research was conducted in the absence of any commercial or financial relationships that could be construed as a potential conflict of interest.
