# Supplementary material for: Senolytic Peptide FOXO4-DRI Selectively Removes Senescent Cells From in vitro Expanded Human Chondrocytes
Source: Front Bioeng Biotechnol. 2021 Apr 29;9:677576. doi: 10.3389/fbioe.2021.677576 (PMC8116695; doi:10.3389/fbioe.2021.677576)
Supplement: Supplementary file 1 [file Table_1.DOCX]

Supplementary Material

# Supplementary Figures and Tables

## Supplementary Figures

**
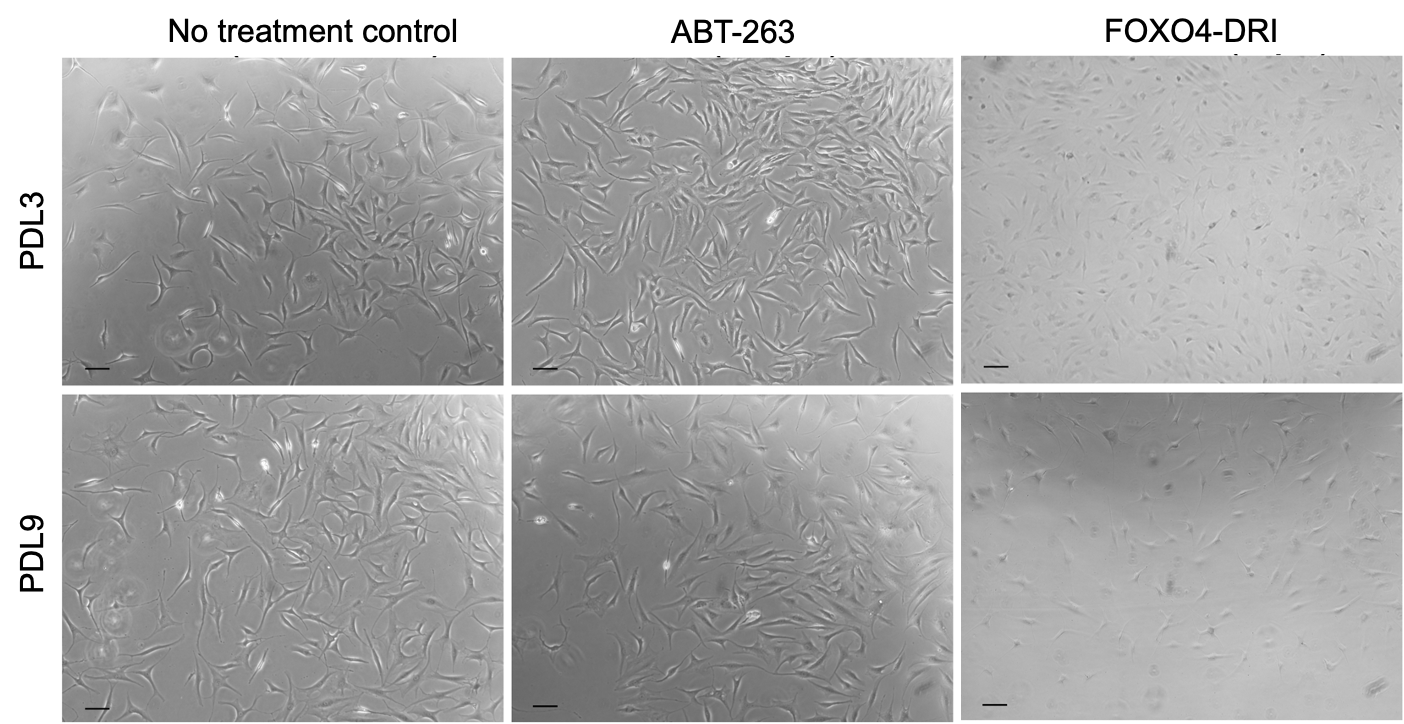
**

**Supplementary Figure S1.** The PDL3 and PDL9 culture were treated with ABT-263 (ABT) or FOXO4-DRI for 5 days. Bar=100μm.

## Supplementary Tables

**Supplementary Table S1.** Full name and abbreviation of gene and protein.

| Full name | Gene | Protein |
| --- | --- | --- |
| Ribosomal protein L13a | *RPL13a* | RPL13A |
| Collagen type II alpha 1 chain | *COL2* | COL2 |
| Aggrecan | *ACAN* | ACAN |
| SRY-Box transcription factor 9 | *SOX9* | SOX9 |
| Matrix metallopeptidase 1 | *MMP1* | MMP1 |
| Matrix metallopeptidase 3 | *MMP3* | MMP3 |
| Matrix metallopeptidase 12 | *MMP12* | MMP12 |
| Matrix metallopeptidase 13 | *MMP13* | MMP13 |
| Interleukin 6 | *IL6* | IL-6 |
| Interleukin 8 | *IL8* | IL-8 |
| Cyclin-dependent kinase inhibitor 2A | *CDKN2A* | p16 |
| Cyclin-dependent kinase inhibitor 1A | *CDKN1A* | p21 |
| Tumor antigen p53 | *TP53* | p53 |
| Osteocalcin | *OCN* | OCN |
| Osteopontin | *OPN* | OPN |
| A disintegrin and metalloproteinase with thrombospondin motifs 5 | *ADAMTS5* | ADAMTS5 |

**Supplementary Table S2.** Primer sequences used for RT-qPCR.

| Gene | Forward primer (5’->3’) | Reverse primer (5’->3’) |
| --- | --- | --- |
| *RPL13a* | CATAGGAAGCTGGGAGCAAG | GCCCTCCAATCAGTCTTCTG |
| *COL2A1* | *GGATGGCTGCACGAAACATACCGG* | *CAAGAAGCAGACCGGCCCTATG* |
| *ACAN* | *AGTCACACCTGAGCAGCATC* | *AGTTCTCAAATTGCATGGGGTGTC* |
| *SOX9* | *GGCGGAGGAAGTCGGTGAAGAA* | *GCTCATGCCGGAGGAGGAGTGT* |
| *MMP13* | *ATGCAGTCTTTCTTCGGCTTAG* | *ATGCCATCGTGAAGTCTGGT* |
| *MMP12* | *GGAATCCTAGCCCATGCTTTT* | *CATTACGGCCTTTGGATCACT* |
| *MMP1* | *AAAATTACACGCCAGATTTGCC* | *GGTGTGACATTACTCCAGAGTTG* |
| *MMP3* | *AGTCTTCCAATCCTACTGTTGCT* | *TCCCCGTCACCTCCAATCC* |
| *ALP* | *ATCTTTGGTCTGGCCCCCATG* | *AGTCCACCATGGAGACATTCTCTC* |
| *OCN* | *TCACACTCCTCGCCCTATTG* | *GAAGAGGAAAGAAGGGTGCC* |
| *OPN* | *TCACCAGTCTGATGAGTCTCACCATTC* | *TAGCATCAGGGTACTGGATGTCAGGTC* |
| *IL6* | *ACTCACCTCTTCAGAACGAATTG* | *CCATCTTTGGAAGGTTCAGGTTG* |
| *IL8* | *ACTGAGAGTGATTGAGAGTGGAC* | *AACCCTCTGCACCCAGTTTTC* |
| *p16* | *CTACTGAGGAGCCAGCGTCT* | *CTGCCCATCATCATGACCT* |
| *p21* | *AGTGGAATTAGCCCTCAGCA* | *CATGGTCCCTGGGTTCTTC* |
| *p53* | *GCCCAACAACACCAGCTCCT* | *CCTGGGCATCCTTGAGTTCC* |
| *ADAMTS5* | *GAACATCGACCAACTCTACTCCG* | *CAATGCCCACCGAACCATCT* |

**Supplementary Table S3. Information of primary antibodies used in this study.**

| Antibody | Company | Catalog | Concentration |
| --- | --- | --- | --- |
| p21 | Abcam | ab218311 | IHC: 1:200; WB: 1:1000 |
| p16 | Abcam | ab108349 | IHC: 1:100; WB: 1:1000 |
| p53 | Abcam | ab26 | WB 1:500 |
| GAPDH | Cell signaling | 5174 | WB: 1:1000 |
| Universal Kit | Vector | PK6200 | - |
| Mouse IgG | Invitrogen | 31450 | 1:1000 |
| Rabbit IgG | Healthcare | NA934-1ML | 1:1000 |
